# University College Hospital and the Royal Ear Hospital

**Published:** 1920-05-01

**Authors:** James E. Arnold

**Affiliations:** Giltspur Street, E.C. 1.


### THE EDITORS LETTER-BOX.

**Correspondence on all subjects is invited, but we cannot in any way be responsible for the opinions expressed by our correspondents, who must give their name and address as a guarantee of good faith, but not necessarily for publication. Correspondents are reminded that brevity of style and conciseness of statement greatly facilitate early insertion.**

### University College Hospital and the Royal Ear Hospital.

*To the Editor of* THE HOSPITAL.

SIR,—In July last, when the question of an amalgamation of the Royal Ear Hospital with a general hospital—viz., University College Hospital—was brought before me, as a member of the Committee of Management and a Life Governor of the former hospital, I fully considered the matter, and, as the result of that consideration, I entirely opposed the transfer to University College Hospital of any of the funds until I was assured that the work of the Royal Ear Hospital would be carried on in its entirety. I went to the Charity Commission with my objection, for I felt that they might have had some jurisdiction in the matter, but the Charity Commission felt uncertain that the Royal Ear Hospital did come within their jurisdiction, though they expressed themselves as being satisfied that the Governors of that hospital would be fully justified in hesitating to proceed with the transfer without an order of the Commission.

What I felt was that the so-called amalgamation was one that would extingush the Royal Ear Hospital to the advantage of University College Hospital in that the latter would become possessed of its assets and freehold with very little guarantee that the patients sent up by subscribers of the Royal Ear Hospital, or by Life Governors of that hospital would receive, as I submit that they should, preferential treatment, and, moreover, no out-patients were to be treated at the Royal Ear Hospital, but only in-patients. In short, it appeared to me to be a scheme for adding beds to University College Hospital, in additional to giving the latter a valuable property and a possible grant from King Edward's Hospital Fund for London.

The following appeared in a paragraph in the *Evening Standard* of March 30, headed "Menaced Hospital. Treasurer's Plea for University College Institution." It speaks for itself :—

"The Hospital was in a state of bankruptcy, and he [Sir Ernest Hatch] and his colleagues would meet at an early date to decide what action was possible.

"Sir Ernest added that there was no more efficient hospital in London. The public did not adequately realise what important functions a hospital performed in the training of medical students. The lamentable shortage of beds was a reproach to our civilisation."

I think this matter is one that calls for some full explanation from the chairman of both hospitals.—I am, Yours faithfully,

JAMES E. ARNOLD.

Giltspur Street, E.C. 1.
April 21, 1920.

